# Progress in the application of 68Ga-Pentixafor PET/CT in lung cancer: a review

**DOI:** 10.3389/or.2026.1797818

**Published:** 2026-04-22

**Authors:** Lisheng Liu, Juxing Zhang

**Affiliations:** 1 Department of Nuclear Medicine, Jiulongpo District People’s Hospital, Chongqing, China; 2 Department of Nuclear Medicine, Chongqing University of Posts and Telecommunications, Chongqing, China

**Keywords:** 68Ga-Pentixafor PET/CT, CXCR4, diagnostic value, lung cancer, molecular imaging, radioligand therapy

## Abstract

68Ga-Pentixafor PET/CT, a molecular imaging technique targeting the C-X-C chemokine receptor 4 (CXCR4), has emerged as a promising tool in the diagnosis and therapeutic evaluation of lung cancer. Lung cancer remains a leading cause of cancer-related mortality worldwide, and accurate imaging modalities are critical for early detection, staging, and treatment monitoring. Current imaging approaches face challenges in differentiating tumor subtypes and assessing tumor biology, which limits personalized treatment strategies. This review systematically summarizes the application of 68Ga-Pentixafor PET/CT across various histological subtypes of lung cancer, including non-small cell lung cancer (NSCLC), small cell lung cancer (SCLC), and rare lung cancer variants. We emphasize the correlation between PET imaging findings and immunohistochemical CXCR4 expression, highlighting the diagnostic value and unique imaging characteristics of this modality. Additionally, the potential role of 68Ga-Pentixafor PET/CT in guiding radioligand therapy is discussed, alongside its utility in tumor staging, therapeutic response assessment, and individualized treatment planning. By integrating recent clinical studies and systematic evaluations, this review aims to elucidate the advantages and limitations of 68Ga-Pentixafor PET/CT, providing theoretical insights and practical guidance for advancing precision medicine in lung cancer management.

## 1 Introduction

Lung cancer is a malignant tumor that originates in the lungs, typically in the cells lining the air passages. It is 1 of the most common and deadliest forms of cancer world wide. There are 2 main types of lung cancer: nonsmall cell lung cancer (NSCLC) and small cell lung cancer (SCLC). NSCLC accounts for about 80%–85% of all lung cancers, while SCLC makes up the remaining 10%–15% (1). Its high incidence and mortality rates can be attributed to various factors, including late-stage diagnosis and the aggressive nature of the disease. The early detection of lung cancer is crucial for improving patient outcomes, as survival rates dramatically decrease with advanced disease stages. Traditional diagnostic methods, such as low-dose computed tomography (LDCT), have shown promise in early detection; however, they are limited by high false-positive rates and the need for further invasive procedures. There is a pressing need for innovative approaches that enhance diagnostic accuracy and facilitate timely intervention (2).

Recent advancements in molecular imaging techniques, particularly the use of positron emission tomography (PET) with specific radiotracers, have opened new avenues for the diagnosis and management of lung cancer. One such radiotracer, 68Ga-Pentixafor, targets the C-X-C chemokine receptor type 4 (CXCR4), which is overexpressed in various malignancies, including lung cancer. The CXCR4/CXCL12 axis is a target of interest for diagnostic imaging applications, as highlighted by studies utilizing [68Ga]Ga-Pentixafor PET in cancer diagnosis and prognosis (3). The ability of 68Ga-Pentixafor PET/CT to non-invasively quantify CXCR4 expression *in vivo* represents a significant advancement in the imaging of lung cancer.

The clinical utility of 68Ga-Pentixafor PET/CT has been increasingly recognized in the context of lung cancer. Studies have demonstrated its effectiveness in differentiating between various lung cancer subtypes, assessing treatment response, and predicting patient prognosis. For instance, the sensitivity and specificity of 68Ga-Pentixafor PET/CT for identifying small cell lung cancer (SCLC) compared to non-small cell lung cancer (NSCLC) have been reported to be high, indicating its potential role in guiding treatment decisions (4). Furthermore, the non-invasive nature of this imaging modality allows for repeated assessments, which is particularly beneficial in monitoring disease progression and treatment efficacy over time.

Despite the promising results, there remains a need for comprehensive evaluations of the diagnostic performance of 68Ga-Pentixafor PET/CT across different lung cancer subtypes and stages. Comparative studies with traditional imaging methods, such as 18F-FDG PET/CT, are essential to fully understand the advantages and limitations of CXCR4-targeted imaging in clinical practice. 68Ga-Pentixafor PET/CT has demonstrated diagnostic utility in various solid tumors, although its sensitivity may be lower compared to [18F]FDG PET/CT, and it holds potential as a tool for CXCR4-targeted theranostics (5).

In summary, the application of 68Ga-Pentixafor PET/CT in lung cancer represents a significant advancement in the field of oncology. By enabling the non-invasive assessment of CXCR4 expression, this imaging modality holds promise for improving early diagnosis, guiding treatment decisions, and ultimately enhancing patient outcomes. Future research should focus on elucidating the full clinical potential of 68Ga-Pentixafor PET/CT, including its role in precision medicine and the development of targeted therapies based on CXCR4 signaling pathways.

## 2 Imaging basis and technical characteristics of 68Ga-Pentixafor PET/CT

### 2.1 Biological role of the CXCR4 receptor and its expression in lung cancer

The C-X-C chemokine receptor type 4 (CXCR4) plays a pivotal role in various biological processes, particularly in cancer biology, where it is implicated in tumor cell migration, invasion, and the regulation of the tumor microenvironment. CXCR4 is a receptor for the chemokine CXCL12 (also known as stromal cell-derived factor-1), which is crucial for directing the movement of cancer cells towards areas of metastasis. Estrogen receptor β (ERβ) promotes non-small cell lung cancer invasion through the circ-TMX4/miR-622/CXCR4 signaling pathway, highlighting the role of CXCR4 expression regulation in tumor progression (6). In lung cancer, studies have shown that the expression levels of CXCR4 correlate with tumor aggressiveness and poor prognosis, highlighting its potential as a therapeutic target. CXCR4 is frequently overexpressed in non-small cell lung cancer (NSCLC), with higher expression observed in advanced stages, although its prognostic value in early-stage NSCLC remains unclear (7). Studies have investigated the expression of JUNB and CXCR4 in circulating tumor cells of lung cancer patients and their prognostic implications, without direct assessment of their roles in epithelial-mesenchymal transition (EMT) (8).

The expression of CXCR4 varies among different histological types of lung cancer, which can provide insights into the malignancy’s biological behavior. For instance, studies have demonstrated that adenocarcinoma often exhibits higher CXCR4 expression compared to squamous cell carcinoma, suggesting that histological subtype may influence the receptor’s role in tumor progression (4). Furthermore, elevated CXCR4 levels have been linked to advanced tumor stages and the presence of metastases, indicating that CXCR4 may serve as a biomarker for disease severity and prognosis in lung cancer patients (9). The differential expression of CXCR4 across lung cancer subtypes and its correlation with clinical outcomes emphasize the need for stratified approaches in targeting this receptor in therapeutic interventions.

To validate the expression of CXCR4 in lung cancer, various techniques such as immunohistochemistry and flow cytometry have been employed. These methods have revealed a substantial presence of CXCR4 in tumor tissues compared to normal lung tissues, reinforcing the receptor’s potential as a therapeutic target (10). Mesenchymal stem cells have been shown to inhibit alveolar epithelial cell apoptosis via the CXCL12/CXCR4 axis in acute respiratory distress syndrome models, though the clinical significance of CXCR4 expression in lung cancer prognosis was not addressed (11). The heterogeneity of CXCR4 expression in lung cancer necessitates further investigation to elucidate its role in tumor biology and to explore its potential as a target for novel therapeutic strategies. Given the receptor’s involvement in critical pathways related to tumor progression and metastasis, targeting CXCR4 may provide a promising avenue for improving treatment outcomes in lung cancer patients.

### 2.2 The radioactive labeling principle of 68Ga-Pentixafor and the mechanism of pet/CT imaging

The chemical structure of 68Ga-Pentixafor is pivotal to its function as a specific ligand for the C-X-C chemokine receptor type 4 (CXCR4), which is often overexpressed in various cancers, including lung cancer. The compound is synthesized through a radiolabeling process that involves the coordination of gallium-68, a positron-emitting radionuclide, to the Pentixafor peptide. This process typically utilizes a bifunctional chelator, such as DOTA (1,4,7,10-tetraazacyclododecane-1,4,7-triacetic acid), which facilitates the stable binding of gallium-68 to the peptide. The high affinity of Pentixafor for CXCR4 allows for effective targeting of tumors that express this receptor, making it a valuable tool in oncological imaging. The specificity of 68Ga-Pentixafor for CXCR4 is supported by its ability to bind to the receptor with high affinity, which is crucial for achieving optimal imaging results in PET/CT scans. Automated radiosynthesis methods have been developed for [68Ga]Ga-Pentixafor and other PET tracers, facilitating their use in imaging various cancers, though specific clinical imaging benefits in heterogeneous tumors such as lung cancer were not detailed (12).

The imaging process using PET/CT with 68Ga-Pentixafor involves a series of steps that ensure accurate detection and quantification of CXCR4 expression in tumors. Initially, patients are injected with the radiolabeled tracer, which subsequently accumulates in tissues expressing CXCR4. The PET imaging technique detects the emitted positrons from the radioactive decay of gallium-68, allowing for the generation of detailed images of the distribution of the tracer within the body. The standardized uptake value (SUV) is a semi-quantitative measure used to assess the uptake of the tracer in tissues, with the maximum standardized uptake value (SUVmax) being particularly important for evaluating tumor activity. The SUVmax provides insight into the metabolic activity of the tumor, as higher values typically correlate with increased receptor expression and, consequently, more aggressive tumor behavior. CXCR4-targeted PET/CT imaging has been compared with 18F-FDG PET/CT and immunohistochemistry in head and neck cancers, focusing on diagnostic performance rather than semi-quantitative assessment for lesion differentiation or treatment monitoring (13).

**FIGURE 1:**
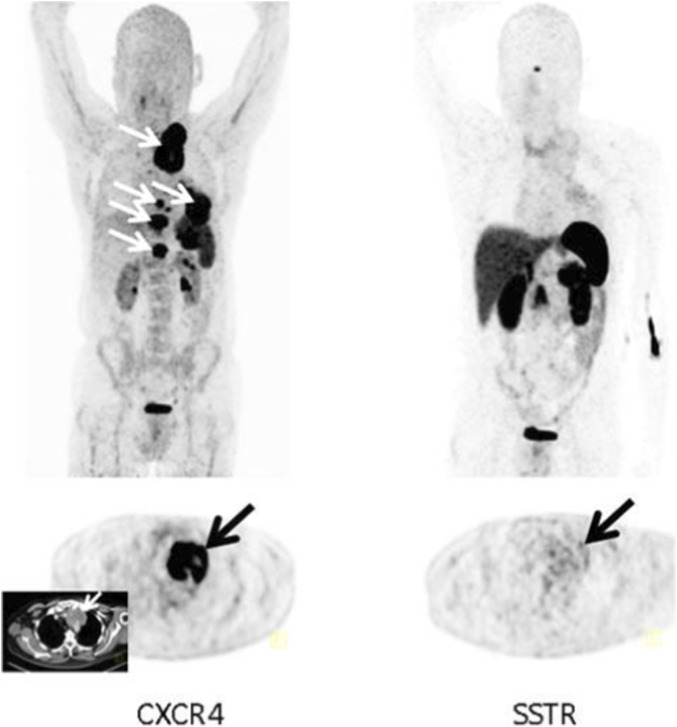
Example of a CXCR4-positive, SSTR-negative SCLC patient. Display of maximum intensity projections (upper row) and transaxial images (lower row) of both CXCR4- and SSTR-directed PET/CT (interval between both scans, 6 days) in a patient with recurrent SCLC (patient #3). Whereas [^68^Ga]Pentixafor-PET demonstrates intense tracer retention in various tumor manifestations including mediastinal lymph nodes, bone and pleural lesions, SSTR-directed PET proves negative (arrows; insert: corresponding contrast-enhanced transaxial CT) (19).

One of the significant advantages of using 68Ga-Pentixafor in PET/CT imaging is its superior imaging contrast and signal-to-noise ratio (SNR), which are particularly pronounced in the context of heterogeneous tumor expression (Figure 1). In lung cancer, where tumor microenvironments can vary significantly, the ability to visualize areas of high CXCR4 expression can aid in treatment planning and prognostication. The high SNR achieved with 68Ga-Pentixafor PET imaging allows for better delineation of tumor boundaries and facilitates the identification of metastatic lesions that may not be as clearly visible with traditional imaging modalities, such as CT or MRI. CXCR4 serves as a biomarker for imaging and targeted radionuclide therapy using 68Ga-Pentixafor and Pentixather, though the impact of enhanced contrast on clinical decision-making was not specifically addressed (14). Automated radiosynthesis protocols for [68Ga]Ga-Pentixafor have been optimized for clinical use, with applications in imaging various cancers, though specific advantages over 18F-FDG PET regarding brown adipose tissue uptake or superiority in lung cancer imaging were not reported (12).

In summary, the unique chemical structure and binding properties of 68Ga-Pentixafor, combined with the advanced imaging capabilities of PET/CT, position this radiotracer as a promising tool in the diagnosis and management of lung cancer. Its ability to provide clear, high-contrast images of CXCR4 expression not only enhances diagnostic accuracy but also supports personalized treatment approaches based on the molecular characteristics of individual tumors.

### 2.3 Comparison analysis with traditional 18F-FDG PET/CT

The application of 18F-FDG PET/CT in lung cancer diagnosis has been widely recognized; however, it is not without limitations. One of the primary drawbacks of 18F-FDG PET/CT is its susceptibility to false positives due to inflammatory processes. [68Ga]Ga-Pentixafor PET imaging has been reviewed for its diagnostic and prognostic applications in various cancers, without specific discussion of misinterpretation of benign lesions as malignant (3). Additionally, 18F-FDG PET/CT may struggle with the identification of low-metabolism tumors, which can be particularly problematic in certain lung cancer subtypes. Small-cell lung cancer (SCLC) has shown higher uptake values on [68Ga]Ga-Pentixafor PET/CT, though the relationship to glucose metabolism levels and detection rates requires further investigation (5). This limitation is critical because accurate staging and treatment planning depend on reliable imaging modalities. In contrast, 68Ga-Pentixafor PET/CT has shown promise in addressing these shortcomings. [68Ga]Ga-Pentixafor PET/CT has been evaluated primarily in multiple myeloma patients for staging and prognosis, without specific data on CXCR4 expression in lung cancer or enhanced detection of low-glucose-avid tumors (15). This receptor-targeted imaging approach allows for better differentiation between malignant and benign lesions, thus potentially reducing the rate of false-positive findings that plague 18F-FDG PET/CT.

**FIGURE 2:**
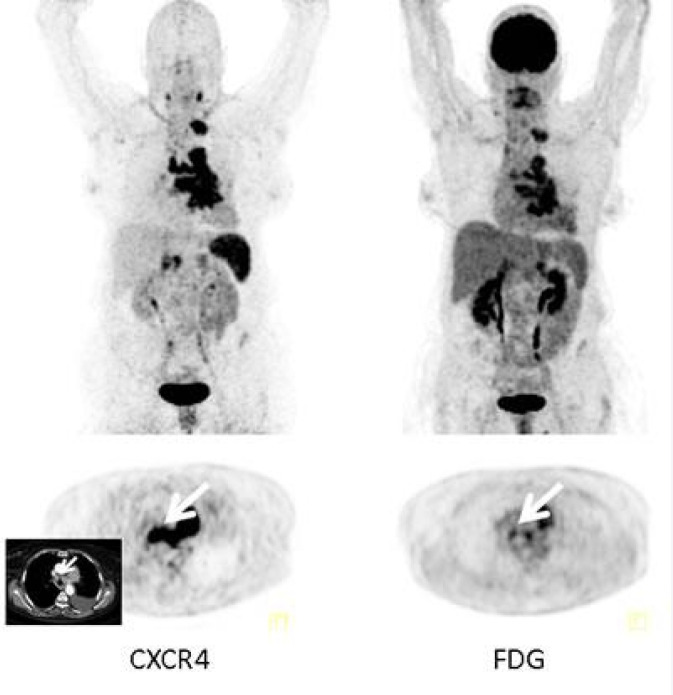
Example of additional value of [^68^Ga]Pentixafor-PET in an SCLC patient. Display of maximum intensity projections (upper row) and transaxial images (lower row) of both CXCR4- and [18F]FDG-PET/CT (interval between both scans, 6 days) in a patient with recurrent SCLC (patient #4). [68Ga]Pentixafor-PET demonstrates more intense tracer retention in various tumor manifestations including mediastinal lymph nodes (arrows; insert: corresponding contrast-enhanced transaxial CT) (19).

In terms of clinical utility, 68Ga-Pentixafor PET/CT has demonstrated higher sensitivity and specificity in certain lung cancer subtypes, such as SCLC and adenocarcinoma (Figure 2). Comparative studies have shown similar uptake patterns between 68Ga-Pentixafor PET/CT and 18F-FDG PET/CT in various tissues, with further research needed to clarify the diagnostic advantages of 68Ga-Pentixafor PET/CT (16). 68Ga-Pentixafor PET/CT has demonstrated efficacy in evaluating Waldenström macroglobulinemia/lymphoplasmacytic lymphoma, with no data supporting its use in lung cancer (17). 68Ga-Pentixafor PET/CT has been utilized in nasopharyngeal carcinoma to assess CXCR4 expression, with potential implications for patient selection in CXCR4-targeted radiotherapy, though its role in lung cancer diagnosis and prognosis was not addressed (18). This correlation emphasizes the importance of integrating receptor-targeted imaging into clinical practice to enhance patient outcomes.

The complementary nature of 18F-FDG PET/CT and 68Ga-Pentixafor PET/CT presents an exciting avenue for future clinical applications. Although 18F-FDG PET/CT remains the standard for metabolic imaging, 68Ga-Pentixafor PET/CT serves as a complementary tool for identifying patients who may benefit from CXCR4-targeted therapies, without evidence supporting dual-modality optimization of diagnosis and treatment planning (13). Combining both imaging techniques may allow clinicians to leverage the strengths of each modality, potentially leading to improved patient management strategies. Comparative studies of 68Ga-Pentixafor PET/CT and 18F-FDG PET/CT in multiple myeloma have focused on staging and prognosis, without evaluation of their combined use for detecting low-metabolism tumors or treatment response assessment (15). As research continues to evolve, the integration of these imaging modalities could pave the way for personalized medicine approaches in lung cancer, where treatment is tailored based on the specific characteristics of the tumor as revealed by advanced imaging techniques.

## 3 Application of 68Ga-Pentixafor PET/CT in different histological subtypes of lung cancer

### 3.1 Small cell lung cancer (SCLC)

Small cell lung cancer (SCLC) is characterized by a significant overexpression of the C-X-C motif chemokine receptor 4 (CXCR4), which plays a crucial role in tumor progression and metastasis. Studies have shown that the standardized uptake value maximum (SUVmax) of 68Ga-Pentixafor, a radiotracer targeting CXCR4, is markedly elevated in SCLC compared to non-small cell lung cancer (NSCLC). Specifically, the mean SUVmax in SCLC has been reported to be significantly higher than that in NSCLC, with values averaging around 10.3, indicating a strong *in vivo* uptake of 68Ga-Pentixafor due to the high density of CXCR4 receptors present in SCLC tumors (4). This elevated expression of CXCR4 not only underscores the aggressive nature of SCLC but also highlights the potential of 68Ga-Pentixafor PET/CT as a diagnostic tool that could aid in differentiating SCLC from other lung cancer subtypes, particularly in clinical settings where accurate staging is critical for treatment planning.

The correlation between imaging findings from 68Ga-Pentixafor PET/CT and immunohistochemical analysis of CXCR4 expression has been a focal point of research. In a study involving 94 lung cancer patients, a significant relationship was established between SUVmax values obtained from PET imaging and the mean fluorescence index (MFI) derived from immunohistochemistry. While the correlation was robust in NSCLC, it was less pronounced in SCLC, potentially due to the unique biological behavior of SCLC and the limited sample size (4). This discrepancy suggests that while 68Ga-Pentixafor PET/CT can effectively visualize CXCR4 expression, further investigation is necessary to fully understand the implications of these imaging biomarkers in SCLC. The ability of 68Ga-Pentixafor PET/CT to provide insights into tumor biology, alongside traditional imaging modalities, could enhance the precision of SCLC staging and treatment response assessments.

The clinical utility of 68Ga-Pentixafor PET/CT extends beyond mere diagnosis; it holds promise for improving the staging and therapeutic management of SCLC. The sensitivity and specificity of this imaging modality for differentiating SCLC from NSCLC have been reported at 85.7% and 78.1%, respectively, demonstrating its potential as a reliable tool in clinical practice (4). Furthermore, the high SUVmax values associated with SCLC suggest that this imaging technique could be instrumental in evaluating treatment response, particularly in the context of CXCR4-targeted therapies. As ongoing research continues to elucidate the role of CXCR4 in tumor biology, the integration of 68Ga-Pentixafor PET/CT into routine clinical workflows may facilitate more personalized approaches to SCLC management, ultimately leading to improved patient outcomes.

In summary, the application of 68Ga-Pentixafor PET/CT in SCLC represents a significant advancement in the field of molecular imaging. Its ability to non-invasively assess CXCR4 expression not only aids in the differentiation of SCLC from other lung cancer subtypes but also enhances the accuracy of staging and treatment response evaluation.This novel PET tracer might serve as an innovative imaging agent for *in vivo* biomarker identification that could result in patient selection for CXCR4-directed treatment, and, eventually, for receptor-radio(drug)peptide therapy (19). As the understanding of CXCR4’s role in tumor biology deepens, the potential for 68Ga-Pentixafor PET/CT to inform therapeutic strategies and improve patient outcomes in SCLC becomes increasingly evident. Future studies should aim to validate these findings in larger cohorts and explore the implications of CXCR4-targeted therapies in the management of SCLC.

### 3.2 Non-small cell lung cancer (NSCLC)

The expression of the C-X-C motif chemokine receptor 4 (CXCR4) in non-small cell lung cancer (NSCLC) varies significantly between its subtypes, particularly adenocarcinoma and squamous cell carcinoma. Recent studies utilizing [68Ga]Ga-Pentixafor PET/CT imaging have demonstrated that CXCR4 expression levels differ markedly between these histological variants, which is critical for understanding their biological behavior and therapeutic responses. In a study involving 94 lung cancer patients, it was found that the mean standardized uptake value (SUVmax) for adenocarcinoma was significantly higher at 8.0 compared to 6.2 for squamous cell carcinoma, indicating a stronger *in vivo* CXCR4 expression in adenocarcinomas (4). This differential expression suggests that adenocarcinomas may have a distinct molecular profile that can be targeted for therapeutic interventions. The PET/CT imaging results correlate well with immunohistochemical analyses, reinforcing the notion that CXCR4 is a viable biomarker for distinguishing between NSCLC subtypes.

The SUVmax, a crucial parameter in PET imaging, not only reflects the level of CXCR4 expression but also serves as a potential prognostic indicator. The significantly higher SUVmax in adenocarcinoma compared to squamous cell carcinoma suggests that adenocarcinomas may exhibit more aggressive behavior or a higher propensity for metastasis, which could influence treatment decisions. Furthermore, the sensitivity and specificity of [68Ga]Ga-Pentixafor PET/CT for differentiating between these two NSCLC subtypes were found to be 87.5% and 71.4%, respectively, with an ROC cutoff SUVmax of 6.7 (4). This highlights the utility of PET imaging in clinical practice, where accurate subtype identification is crucial for tailoring treatment strategies.

The correlation between imaging parameters and histological subtypes extends beyond mere differentiation; it also encompasses the diagnostic sensitivity and specificity of imaging findings. In the context of NSCLC, the ability of [68Ga]Ga-Pentixafor PET/CT to accurately identify adenocarcinoma versus squamous cell carcinoma enhances its role as a diagnostic tool. The study indicated that a significant correlation exists between SUVmax and mean fluorescence index (MFI) values in both adenocarcinoma and squamous cell carcinoma, suggesting that PET imaging can effectively reflect the underlying biology of these tumors (4). However, the correlation was not as robust in small cell lung cancer (SCLC) and neuroendocrine neoplasms, indicating the complexity of tumor biology and the need for further research in these areas.

In conclusion, the differential expression of CXCR4 in NSCLC subtypes, as evidenced by [68Ga]Ga-Pentixafor PET/CT imaging, underscores the importance of molecular imaging in the management of lung cancer. The higher SUVmax in adenocarcinoma not only provides insights into the biological behavior of this subtype but also opens avenues for targeted therapies that exploit CXCR4 as a therapeutic target. As our understanding of CXCR4’s role in lung cancer deepens, the integration of advanced imaging techniques like PET/CT into clinical practice will likely enhance diagnostic accuracy and improve patient outcomes through more personalized treatment approaches.

### 3.3 Rare lung cancers and pulmonary metastases

Rare lung cancer subtypes, such as hemangioendothelioma, sarcomatoid carcinoma, and hemangiopericytoma, present unique challenges in diagnosis and treatment due to their uncommon nature and atypical presentations. Recent studies utilizing 68Ga-Pentixafor PET/CT imaging have highlighted the expression of C-X-C chemokine receptor type 4 (CXCR4) in these malignancies, which is crucial for understanding their biological behavior and potential therapeutic targets. For instance, a study involving six patients with suspected lung masses demonstrated that 68Ga-Pentixafor PET/CT could effectively visualize increased CXCR4 expression, correlating with histopathological findings. The maximum standardized uptake values (SUVmax) observed were notably high, particularly in hemangioendothelioma, which showed an SUVmax of 13.0, indicating a robust expression of CXCR4. This suggests that CXCR4-targeted imaging could play a significant role in the non-invasive assessment of rare lung cancer variants, potentially guiding therapeutic decisions and improving patient outcomes (20).

The potential application of 68Ga-Pentixafor PET/CT in differentiating between primary tumors and metastatic lesions is another critical aspect of its utility in the realm of rare lung cancers. The ability of this imaging modality to accurately identify and characterize lesions can significantly impact treatment planning. In a cohort of patients with various malignancies, including lung cancers, 68Ga-Pentixafor PET/CT demonstrated comparable results to traditional 18F-FDG PET/CT in detecting primary tumors and metastases. Notably, it was able to identify brain metastases that were missed by 18F-FDG PET/CT, underscoring its potential superiority in certain contexts. This capability is particularly beneficial in cases where the primary tumor is not well-defined, or when distinguishing between benign and malignant lesions is challenging. 68Ga-Pentixafor PET/CT has demonstrated diagnostic value comparable to 18F-FDG PET/CT in non-small cell lung cancer (NSCLC), visualizing primary tumors and most metastases, though its specificity for rare lung cancers has not been specifically established (21).

Despite the promising findings associated with 68Ga-Pentixafor PET/CT, challenges remain in correlating imaging results with histopathological data. The relationship between imaging characteristics and the underlying tumor biology can be complex, particularly in rare lung cancer subtypes where histological features may not always align with imaging findings. For example, while increased CXCR4 expression correlates with higher SUVmax values, discrepancies can occur due to variations in tumor microenvironment or receptor heterogeneity. Moreover, the diagnostic challenges posed by the rarity of these cancers can lead to difficulties in establishing robust correlations between imaging and pathology. A study that examined 142 patients with various solid tumors, including lung cancers, found significant variability in CXCR4 expression across different histological types, highlighting the need for further research to elucidate these relationships. Understanding the nuances of these correlations is essential for optimizing the diagnostic and therapeutic applications of 68Ga-Pentixafor PET/CT in clinical practice (22).

In conclusion, the integration of 68Ga-Pentixafor PET/CT into the diagnostic workflow for rare lung cancers and pulmonary metastases represents a significant advancement in the field of oncological imaging. Its ability to non-invasively assess CXCR4 expression not only enhances the understanding of tumor biology but also aids in differentiating between primary and metastatic lesions. However, the challenges related to correlating imaging findings with histopathological data necessitate ongoing research to refine the use of this imaging modality and improve patient management strategies. As the field evolves, the potential for 68Ga-Pentixafor PET/CT to inform targeted therapies for rare lung cancers remains a promising avenue for future exploration (4).

## 4 Clinical diagnostic value and efficacy monitoring of 68Ga-Pentixafor PET/CT

### 4.1 Staging and lesion detection in lung cancer

The application of 68Ga-Pentixafor PET/CT in lung cancer has demonstrated significant advancements in the detection of both primary and metastatic lesions, primarily due to its targeting of the C-X-C motif chemokine receptor 4 (CXCR4). This receptor is known to play a crucial role in tumor progression and metastasis, making it a valuable target for imaging in malignancies, including lung cancer. Studies have shown that 68Ga-Pentixafor PET/CT can effectively identify lesions in various types of lung cancer, including small-cell lung cancer (SCLC) and non-small-cell lung cancer (NSCLC) subtypes such as adenocarcinoma and squamous cell carcinoma. The systematic review of existing literature indicates that this imaging modality not only enhances the visualization of tumors but also provides insights into the biological behavior of the cancer through the assessment of CXCR4 expression levels (3). By evaluating the uptake of 68Ga-Pentixafor, clinicians can gain critical information regarding the aggressiveness of the tumor and its potential for metastasis, which is essential for accurate staging and treatment planning.

When compared to traditional imaging techniques such as 18F-FDG PET/CT, 68Ga-Pentixafor PET/CT has shown varying degrees of effectiveness in lesion detection and clinical staging accuracy. While 18F-FDG PET/CT remains the gold standard for metabolic imaging of tumors, it does not specifically target the CXCR4 receptor, which can limit its ability to detect certain malignancies that exhibit low FDG avidity. In contrast, 68Ga-Pentixafor PET/CT has been noted to provide better visualization in specific cases, particularly in tumors that express high levels of CXCR4, which can lead to improved detection rates and more precise staging of lung cancer. However, it is important to note that the diagnostic performance of 68Ga-Pentixafor PET/CT may not universally surpass that of 18F-FDG PET/CT across all tumor types, as some malignancies demonstrate minimal or absent CXCR4 expression (5). Therefore, while 68Ga-Pentixafor PET/CT offers a promising alternative, its role should be considered complementary to traditional imaging methods, particularly in the context of precision medicine.

The uniformity of CXCR4 expression across multiple lesions in patients with lung cancer is another critical factor that influences treatment decisions. The assessment of CXCR4 expression via 68Ga-Pentixafor PET/CT can reveal the heterogeneity of receptor expression within a tumor or between metastatic sites, which may have implications for targeted therapies. For instance, a consistent high expression of CXCR4 across multiple lesions could indicate a more aggressive disease and suggest a favorable response to CXCR4-targeted therapies. Conversely, heterogeneous expression may complicate treatment decisions, as some lesions may not respond to therapies aimed at CXCR4. Understanding the distribution and intensity of CXCR4 expression through imaging can thus guide clinicians in tailoring treatment approaches, potentially leading to more personalized and effective management of lung cancer patients (23). Overall, the integration of 68Ga-Pentixafor PET/CT into clinical practice holds promise for enhancing the accuracy of lung cancer staging and lesion detection, ultimately contributing to improved patient outcomes.

### 4.2 Efficacy evaluation and prognostic prediction

The assessment of treatment response and prognostic prediction in lung cancer using 68Ga-Pentixafor PET/CT is significantly enhanced by imaging parameters such as the maximum standardized uptake value (SUVmax) and tumor-to-background ratio (TBR). SUVmax is a quantitative measure of radiotracer uptake in the tumor, providing a direct correlation with the metabolic activity of the cancer cells. Studies have shown that higher SUVmax values are often associated with more aggressive tumor phenotypes and can predict treatment response. For instance, in a cohort of lung cancer patients, a significant correlation was observed between high SUVmax values and increased CXCR4 expression, suggesting that tumors with elevated CXCR4 levels may be more metabolically active and responsive to therapies targeting this receptor (4). Furthermore, the TBR, which compares the uptake in the tumor to that in the surrounding normal tissue, serves as an important parameter for evaluating the specificity of the imaging and the degree of tumor involvement. A higher TBR indicates a clearer delineation of the tumor from the background, which is crucial for accurate treatment monitoring and prognostic assessment. These imaging parameters not only facilitate the evaluation of therapeutic efficacy but also help in stratifying patients based on their likely response to treatment, thereby guiding personalized therapeutic approaches.

The potential of 68Ga-Pentixafor PET/CT in monitoring the efficacy of various treatment modalities such as radiotherapy, chemotherapy, and targeted therapies is noteworthy. This imaging technique allows for real-time assessment of CXCR4 expression levels, which can fluctuate with treatment. For example, in patients undergoing chemotherapy, a decrease in SUVmax post-treatment can indicate a positive therapeutic response, while stable or increasing values may suggest treatment resistance or disease progression (3). Moreover, the ability to visualize changes in CXCR4 expression in response to therapy provides insights into the tumor microenvironment and its interaction with therapeutic agents. This dynamic monitoring capability is particularly advantageous in lung cancer, where the heterogeneity of tumor response can complicate treatment decisions. The integration of 68Ga-Pentixafor PET/CT into routine clinical practice could therefore enhance the precision of treatment monitoring, allowing for timely modifications to therapeutic strategies based on individual patient responses.

The expression level of CXCR4 has emerged as a significant prognostic marker in lung cancer, influencing both survival outcomes and recurrence risk. High levels of CXCR4 expression are often associated with poor prognosis, as they correlate with increased tumor aggressiveness, metastatic potential, and resistance to conventional therapies. CXCR4-directed PET/CT imaging has been evaluated for its applications in solid tumors, including imaging characteristics and suitability for CXCR4-targeted therapies, without assessment of CXCR4 expression levels in relation to survival or recurrence (22). This relationship underscores the importance of CXCR4 as a target for both diagnostic imaging and therapeutic interventions. By utilizing 68Ga-Pentixafor PET/CT, clinicians can not only assess the expression of CXCR4 in real-time but also stratify patients based on their risk profiles, thereby tailoring treatment plans to improve outcomes. The prognostic implications of CXCR4 expression highlight its role in the evolving landscape of personalized medicine in oncology, particularly in lung cancer management, where accurate prognostication is crucial for optimizing patient care.

### 4.3 Potential target evaluation for radioligand therapy

The application of 68Ga-Pentixafor PET/CT for assessing the suitability of CXCR4-targeted radioligand therapy has garnered significant attention in recent studies. CXCR4, a chemokine receptor implicated in tumor progression and metastasis, is overexpressed in various malignancies, including lung cancer. The utilization of 68Ga-Pentixafor PET/CT allows for the *in vivo* quantification of CXCR4 receptor expression, which can serve as a critical biomarker for determining the appropriateness of CXCR4-targeted therapies. For instance, a study involving 94 lung cancer patients demonstrated that 79.8% had non-small cell lung cancer (NSCLC), with significant variations in CXCR4 expression across different histological subtypes. The study found that small cell lung cancer (SCLC) exhibited the highest uptake of 68Ga-Pentixafor, suggesting a potential therapeutic window for CXCR4-targeted treatments in this subgroup (4). Moreover, the correlation between PET-derived standardized uptake values (SUVmax) and immunohistochemistry (IHC) findings supports the notion that 68Ga-Pentixafor can effectively guide patient selection for CXCR4-targeted radioligand therapy, thereby enhancing personalized treatment approaches.

Initial studies have also explored the role of imaging in screening patients for CXCR4-targeted therapies and predicting treatment efficacy. The sensitivity and specificity of 68Ga-Pentixafor PET/CT in differentiating SCLC from NSCLC were reported to be 85.7% and 78.1%, respectively, indicating its potential utility in clinical decision-making (4). Furthermore, the imaging modality has demonstrated a strong correlation between CXCR4 receptor density and tumor burden, suggesting that patients with a higher number of CXCR4-avid lesions may be more likely to benefit from targeted therapies. This correlation is particularly relevant in the context of advanced lung cancer, where traditional therapeutic options may be limited. The ability to visualize and quantify CXCR4 expression non-invasively positions 68Ga-Pentixafor PET/CT as a valuable tool for both patient stratification and monitoring treatment response.

Looking ahead, the integration of CXCR4 antagonists with radioligand therapies presents a promising strategy for enhancing therapeutic outcomes in lung cancer. The combination of 68Ga-Pentixafor PET imaging with CXCR4-targeted radioligand therapy could potentially overcome resistance mechanisms that tumors develop against conventional treatments. Preclinical studies have indicated that the simultaneous targeting of CXCR4 with radioligands, such as 177Lu-labeled agents, alongside CXCR4 antagonists, may synergistically inhibit tumor growth and improve survival rates (24). This approach not only aims to enhance the efficacy of radioligand therapy but also addresses the tumor microenvironment’s role in facilitating cancer progression. As research continues to elucidate the complexities of CXCR4 signaling pathways and their interactions with the tumor microenvironment, the development of combination therapies that leverage both imaging and targeted treatment modalities could significantly advance the field of personalized oncology, particularly in lung cancer management. Overall, the potential of 68Ga-Pentixafor PET/CT in evaluating CXCR4 as a therapeutic target underscores its relevance in the evolving landscape of cancer treatment strategies.

## 5 Challenges and limitations of 68Ga-Pentixafor PET/CT in lung cancer research

### 5.1 Research design and sample heterogeneity issues

The current landscape of research utilizing 68Ga-Pentixafor PET/CT in lung cancer is characterized by a predominance of single-center studies with relatively small sample sizes, which presents significant challenges in terms of generalizability and the robustness of findings. Many studies focus on specific lung cancer subtypes, such as small cell lung cancer (SCLC) and non-small cell lung cancer (NSCLC), but the limited number of patients involved restricts the ability to draw definitive conclusions regarding the efficacy and diagnostic performance of 68Ga-Pentixafor across the broader spectrum of lung cancer types. For instance, one study reported a total of 94 lung cancer patients, where 79.8% were diagnosed with NSCLC, while only a small fraction represented SCLC and neuroendocrine tumors (4). This imbalance in sample sizes among different lung cancer subtypes can lead to skewed statistical analyses and may compromise the validity of the results. Furthermore, the lack of large-scale, multicenter validation studies exacerbates the issue, as findings from smaller studies may not be replicable in larger, more diverse populations. The absence of multicenter trials also limits the diversity of patient demographics and tumor characteristics, which are crucial for understanding the full potential of 68Ga-Pentixafor PET/CT in various clinical scenarios.

Moreover, the inherent heterogeneity of lung cancer itself complicates the interpretation of results derived from these studies. Lung cancer is a complex disease with multiple histological subtypes, each exhibiting unique biological behaviors and responses to treatment. The differences in CXCR4 receptor expression, as evidenced by varying standardized uptake values (SUVmax) across different subtypes, highlight the necessity for a more nuanced approach to research design (4). For example, studies have shown that SCLC tends to exhibit higher CXCR4 expression compared to NSCLC subtypes such as adenocarcinoma and squamous cell carcinoma, which can influence the imaging outcomes and therapeutic implications associated with 68Ga-Pentixafor PET/CT (4). This variability necessitates careful consideration in the design of future studies to ensure adequate representation of all lung cancer subtypes, thus enhancing the statistical power and reliability of the findings.

In light of these challenges, it is imperative for future research to adopt a more standardized approach, integrating multicenter collaborations that can facilitate larger sample sizes and more representative patient populations. Such studies should also strive to include a comprehensive range of lung cancer subtypes, thereby allowing for more robust statistical analyses and clearer insights into the diagnostic and prognostic capabilities of 68Ga-Pentixafor PET/CT. Additionally, the implementation of standardized imaging protocols and outcome measures will be essential in minimizing variability and enhancing the comparability of results across studies. Ultimately, addressing these issues of research design and sample heterogeneity will be crucial in establishing the role of 68Ga-Pentixafor PET/CT as a valuable tool in the management of lung cancer, paving the way for more personalized and effective therapeutic strategies.

### 5.2 Standardization and quantification challenges of imaging techniques

The application of 68Ga-Pentixafor PET/CT in lung cancer imaging faces significant challenges related to the standardization of measurement protocols, particularly concerning the maximum standardized uptake value (SUVmax) and the time windows for scanning. SUVmax serves as a critical metric for evaluating the metabolic activity of tumors, yet discrepancies in its determination can arise from variations in imaging protocols, patient preparation, and the timing of image acquisition post-injection of the radiotracer. Studies have reported that the SUVmax can be influenced by factors such as the patient’s body mass index, the specific PET/CT scanner used, and the timing of the scan relative to the radiotracer’s peak uptake. A systematic review reported that different tumor types show varying uptake patterns of [68Ga]Ga-Pentixafor, though it did not specifically address the need for tailored scanning protocols to optimize SUVmax measurements (3). Furthermore, the optimal time window for imaging post-injection can vary, with some studies suggesting that earlier scans may yield higher SUVmax values due to the rapid uptake of the radiotracer by tumor cells, while others advocate for later scans to allow for better differentiation between tumor and background signals. This inconsistency can complicate the interpretation of results and hinder the establishment of standardized protocols that are essential for multicenter studies and clinical applications.

Moreover, the lack of uniformity in post-imaging processing and quantitative analysis methods further exacerbates the challenges associated with the use of 68Ga-Pentixafor PET/CT in lung cancer. Variations in image reconstruction algorithms, the use of different software for quantification, and the absence of standardized criteria for defining positive uptake can lead to significant discrepancies in reported outcomes. For example, the interpretation of PET images often relies on subjective assessments, which may vary among radiologists, resulting in inconsistent reporting of tumor avidity and response to therapy. Studies have demonstrated significant correlations between *in vivo* [68Ga]Ga-Pentixafor uptake and *ex vivo* CXCR4 expression, without specific mention of the impact of imaging protocol standardization on data interpretation (4). Furthermore, the integration of advanced imaging techniques, such as hybrid PET/MRI, introduces additional complexity, as the fusion of modalities requires precise alignment and calibration to ensure accurate quantification of tumor uptake.

To address these challenges, there is a pressing need for the development of standardized guidelines for the use of 68Ga-Pentixafor PET/CT in lung cancer imaging. Establishing consensus on optimal imaging protocols, including the timing of scans, patient preparation, and post-processing techniques, is crucial for enhancing the reproducibility and reliability of results. Additionally, multicenter collaborations and large-scale studies are essential to validate these protocols and facilitate the establishment of reference values for SUVmax across different tumor types. By standardizing these methodologies, researchers and clinicians can improve the diagnostic accuracy of 68Ga-Pentixafor PET/CT, thereby enhancing its utility in the personalized management of lung cancer and potentially improving patient outcomes. [68Ga]Ga-Pentixafor PET/CT has diagnostic utility in solid tumors and potential as a theranostic tool for CXCR4-targeted therapies, though the role of standardized imaging protocols and quantitative analysis in advancing these therapies was not specifically discussed (5).

### 5.3 Dynamic changes and heterogeneity of CXCR4 expression

The expression of C-X-C motif chemokine receptor 4 (CXCR4) in tumors is not static; it exhibits dynamic changes influenced by the tumor microenvironment and therapeutic interventions. This temporal and spatial heterogeneity of CXCR4 expression is crucial in understanding tumor behavior and therapeutic responses. Studies have evaluated CXCR4 expression and imaging in esophageal cancer, without specific investigation of its fluctuation due to external stimuli or in lung cancer (25). Meta-analyses have assessed the clinical and prognostic significance of CXCR4 expression in lung cancer, without specific evaluation of TGF-β regulation or its role in EMT and metastasis (9). Additionally, the interaction between cancer cells and surrounding stromal cells, such as cancer-associated fibroblasts, can further enhance CXCR4 signaling, leading to increased tumor invasiveness and resistance to therapies (10). This dynamic nature of CXCR4 expression underscores the importance of considering both the temporal and spatial context of its expression when evaluating treatment efficacy and disease progression in lung cancer patients.

The discrepancies between imaging and histological assessments of CXCR4 expression also raise important clinical considerations. Imaging studies have demonstrated that [68Ga]Ga-Pentixafor PET/CT results correlate well with immunohistochemical staining for CXCR4, confirming target specificity (26). This lack of concordance can arise from several factors, including the inherent limitations of imaging techniques in capturing the full extent of receptor expression, as well as the influence of the tumor microenvironment on receptor availability and accessibility. [68Ga]Ga-Pentixafor PET/CT has been applied in the subtyping of primary aldosteronism, with no reported data on false-negative results related to receptor internalization or downregulation in malignancies (27). CXCR4-targeted PET imaging in breast cancer has been correlated with tumor grade, proliferation index, and triple-negative status, without specific discussion on the impact of immune cell CXCR4 expression on imaging interpretation (28). Clinically, this discrepancy emphasizes the need for a multimodal approach that combines imaging, histological evaluation, and clinical parameters to accurately assess CXCR4 expression and its implications for treatment strategies in lung cancer patients.

In summary, the dynamic changes in CXCR4 expression and the heterogeneity observed between imaging and histological assessments underscore the complexity of targeting this receptor in lung cancer. Understanding these factors is essential for optimizing therapeutic strategies that aim to exploit CXCR4 as a target for treatment and for accurately predicting patient outcomes based on CXCR4 expression profiles. Future research should focus on integrating imaging and histological techniques to provide a more comprehensive understanding of CXCR4 dynamics in the context of lung cancer and its treatment.

### 5.4 Integration with other molecular imaging techniques

The integration of [68Ga]Ga-Pentixafor PET/CT with other molecular imaging modalities, particularly PET/MRI, has shown promising potential in enhancing diagnostic accuracy for lung cancer. The combination of PET and MRI leverages the strengths of both imaging techniques, where PET provides functional information regarding metabolic activity through radiotracer uptake, while MRI offers high-resolution anatomical details and superior soft tissue contrast. This multimodal approach can significantly improve the visualization of lung lesions, especially in complex anatomical regions where precise delineation is crucial for accurate diagnosis and treatment planning. Studies have demonstrated that the use of [68Ga]Ga-Pentixafor PET/MRI can enhance the detection of CXCR4 expression in lung tumors, which is pivotal for assessing tumor behavior and therapeutic targeting. Systematic reviews have discussed the applications of [68Ga]Ga-Pentixafor PET/CT and PET/MRI in staging and treatment response evaluation across various cancers, noting their potential in personalized diagnostics, though specific improvements in lesion characterization and tumor microenvironment understanding were not emphasized (3). Moreover, the ability of MRI to provide functional imaging through techniques such as diffusion-weighted imaging (DWI) can complement the metabolic information obtained from PET, potentially leading to more accurate assessments of tumor aggressiveness and treatment response.

Furthermore, the cross-validation of imaging results through multimodal imaging techniques can enhance the reliability of diagnostic outcomes. By employing multiple imaging modalities, clinicians can corroborate findings from [68Ga]Ga-Pentixafor PET/CT with those from other techniques, such as [18F]FDG PET/CT, which is the standard in oncological imaging. For instance, while [68Ga]Ga-Pentixafor PET/CT may demonstrate lower overall lesion detectability compared to [18F]FDG PET/CT, it has shown superior specificity in certain contexts, such as in differentiating between malignant and benign lesions in cases where [18F]FDG uptake is equivocal (21). This cross-validation is particularly beneficial in complex cases of lung cancer, where distinguishing between tumor recurrence and post-treatment changes can be challenging. The integration of [68Ga]Ga-Pentixafor PET with other imaging modalities provides a more comprehensive view of the tumor’s characteristics, allowing for better-informed clinical decisions regarding management and therapeutic interventions.

Moreover, the potential of multimodal imaging extends to the exploration of multi-target imaging approaches, wherein different radiotracers can be used simultaneously to assess various biological pathways within the same tumor. This strategy can provide insights into tumor heterogeneity and the expression of multiple biomarkers, which may influence treatment responses. Studies have focused on [68Ga]Ga-Pentixafor PET/CT for CXCR4 receptor imaging in lung cancer, without evaluation of combined imaging with [18F]FDG or assessment of glucose metabolism (4). Such integrative approaches are particularly relevant in lung cancer, where diverse histological subtypes may exhibit varying receptor expressions and metabolic profiles. As research continues to evolve, the integration of [68Ga]Ga-Pentixafor PET/CT with other molecular imaging techniques is likely to establish new paradigms in the diagnosis, staging, and treatment monitoring of lung cancer, ultimately contributing to the advancement of precision medicine in oncology.

### 5.5 Ethical, safety, and cost-effectiveness considerations

The safety assessment of radiopharmaceuticals, particularly in the context of [68Ga]Ga-Pentixafor PET/CT, is paramount given the increasing application of this imaging modality in lung cancer and other malignancies. The potential side effects associated with the use of [68Ga]Ga-Pentixafor primarily stem from its radioactive nature. Studies have indicated that this radiotracer has a favorable safety profile, with minimal acute adverse effects reported in patients undergoing PET imaging. Systematic reviews have focused on the diagnostic and prognostic applications of [68Ga]Ga-Pentixafor PET in cancer, without detailed reporting on patient complications or adverse effects (3). Furthermore, the effective radiation dose associated with [68Ga]Ga-Pentixafor PET/CT is comparable to that of conventional imaging techniques, such as CT scans, which mitigates concerns regarding excessive radiation exposure. However, it is essential for healthcare providers to remain vigilant and monitor patients for any delayed reactions, especially in populations with pre-existing conditions or those undergoing concurrent therapies. The ethical implications of using radiopharmaceuticals also necessitate informed consent, where patients must be adequately educated about the benefits, risks, and alternatives to this imaging modality. This ensures that patients can make well-informed decisions regarding their diagnostic and therapeutic pathways, promoting patient autonomy and ethical compliance in clinical practice.

In addition to safety considerations, the economic analysis of [68Ga]Ga-Pentixafor PET/CT plays a critical role in its clinical adoption and accessibility. The cost-effectiveness of this imaging technique must be evaluated against its diagnostic benefits, particularly in the context of lung cancer, where accurate staging and treatment response assessment are crucial for optimizing patient outcomes. Recent studies have evaluated [68Ga]Ga-Pentixafor PET/CT for CXCR4 receptor imaging in lung cancer, without addressing cost-effectiveness or economic impact (4). Moreover, the ability of [68Ga]Ga-Pentixafor PET/CT to identify CXCR4 expression, which is linked to tumor aggressiveness and treatment resistance, can facilitate timely intervention and potentially enhance survival rates, further justifying its economic value. However, access to this advanced imaging technique remains a concern, particularly in resource-limited settings where healthcare infrastructure may not support the high costs associated with radiopharmaceuticals. Addressing these accessibility issues is imperative to ensure equitable healthcare delivery, and strategies such as health insurance coverage, government subsidies, or collaborative research initiatives could help mitigate financial barriers. Ultimately, the integration of [68Ga]Ga-Pentixafor PET/CT into clinical practice requires a balanced consideration of ethical, safety, and economic factors to maximize its benefits for patients with lung cancer and other malignancies.

## 6 Future development directions and research prospects

### 6.1 The need for multicenter large sample clinical trials

The application of 68Ga-Pentixafor PET/CT in lung cancer diagnosis and treatment necessitates the establishment of standardized imaging acquisition and analysis protocols. As the utility of 68Ga-Pentixafor PET/CT has been demonstrated in various malignancies, including lung cancer, it is crucial to develop consistent methodologies that can be replicated across different clinical settings. Standardization will not only enhance the reliability of imaging results but also facilitate comparisons across multicenter studies, thereby strengthening the evidence base for clinical applications. The heterogeneity observed in existing studies, such as variations in patient demographics, tumor types, and imaging protocols, underscores the need for unified guidelines that can help mitigate biases and improve the generalizability of findings (3). Furthermore, the implementation of standardized imaging protocols will aid in the accurate assessment of CXCR4 expression levels across different lung cancer subtypes, which is vital for evaluating the therapeutic potential of CXCR4-targeted radioligand therapies (4).

In addition to standardization, it is imperative to validate the clinical efficacy of 68Ga-Pentixafor PET/CT in lung cancer diagnosis and treatment through large-scale multicenter trials. Studies have demonstrated CXCR4 receptor overexpression in rare pulmonary malignancies using 68Ga-pentixafor PET/CT, without specific data on sensitivity and specificity in SCLC or NSCLC subtypes (20). For instance, a recent study highlighted the high sensitivity (85.7%) and specificity (78.1%) of 68Ga-Pentixafor PET/CT in differentiating SCLC from NSCLC, indicating its potential role in clinical decision-making (4). By conducting multicenter trials, researchers can gather extensive data that will not only confirm these findings but also explore the imaging agent’s ability to predict treatment responses and outcomes in a diverse patient population. The diagnostic utility of [68Ga]Ga-Pentixafor PET/CT in solid tumors, including lung cancer, has been evaluated, though its role in guiding treatment selection amidst evolving therapeutic landscapes requires further investigation (5).

Moreover, multicenter studies can facilitate the exploration of the theranostic potential of 68Ga-Pentixafor PET/CT, which combines diagnostic imaging with targeted therapy. The ability to identify patients who may benefit from CXCR4-targeted treatments can significantly improve personalized medicine approaches in lung cancer care (22). As such, establishing multicenter collaborations that focus on the application of 68Ga-Pentixafor PET/CT in lung cancer will be instrumental in advancing our understanding of its clinical utility and optimizing its use in routine practice. This collaborative effort will also promote the sharing of best practices and foster innovation in imaging techniques, ultimately enhancing patient outcomes in lung cancer management.

In conclusion, the need for multicenter large sample clinical trials is paramount to establish standardized imaging protocols and validate the clinical efficacy of 68Ga-Pentixafor PET/CT in lung cancer. By addressing these issues, the medical community can ensure that this promising imaging modality is effectively integrated into clinical practice, thereby improving diagnostic accuracy and treatment outcomes for lung cancer patients.

### 6.2 Radioligand therapy (RLT) and personalized treatment strategies

Radioligand therapy (RLT) has emerged as a promising approach in the management of lung cancer, particularly through the targeting of the C-X-C motif chemokine receptor 4 (CXCR4). The expression of CXCR4 is notably elevated in various malignancies, including non-small cell lung cancer (NSCLC) and small cell lung cancer (SCLC), which underscores the potential for personalized treatment strategies based on CXCR4 expression levels. Recent studies utilizing 68Ga-Pentixafor PET/CT have demonstrated a significant correlation between *in vivo* CXCR4 receptor expression and tumor burden, suggesting that RLT can be tailored according to the specific CXCR4 expression profiles of individual tumors. In a study of 94 lung cancer patients, 79.8% represented NSCLC cases, with all lung cancer types showing increased CXCR4 expression on PET and flow cytometry; SCLC exhibited higher expression levels compared to NSCLC subtypes (4). This information can be pivotal for designing precision RLT protocols that optimize therapeutic outcomes by selecting patients who are most likely to benefit from CXCR4-targeted therapies. By leveraging the imaging capabilities of 68Ga-Pentixafor PET/CT, clinicians can not only assess tumor aggressiveness but also tailor RLT to enhance efficacy and minimize unnecessary toxicity.

Furthermore, the integration of RLT with immunotherapy and targeted therapies represents an innovative frontier in lung cancer treatment. The CXCR4/CXCL12 axis plays a crucial role in tumor progression and metastasis, and its blockade has been shown to enhance the efficacy of various therapeutic modalities. Combining CXCR4 antagonists with radioligand therapy has demonstrated synergistic effects in preclinical models, resulting in improved tumor control, though evidence for reduced metastasis is not directly reported (24). This combination strategy is particularly relevant in the context of triple-negative breast cancer (TNBC), where the interplay between cancer-associated fibroblasts (CAFs) and tumor cells is mediated by CXCR4 signaling. By disrupting this interaction, researchers have observed enhanced therapeutic responses, suggesting that similar approaches could be beneficial in lung cancer management. Moreover, the potential for using RLT in conjunction with immune checkpoint inhibitors could further enhance the immune response against tumors, providing a multifaceted approach to treatment that addresses both the tumor microenvironment and systemic immunity.

In summary, the advancement of personalized treatment strategies in lung cancer through RLT and the targeting of CXCR4 offers a promising avenue for improving patient outcomes. The ability to tailor therapies based on the specific molecular characteristics of tumors, as demonstrated through the use of 68Ga-Pentixafor PET/CT, allows for more effective and individualized treatment plans. As research continues to explore the synergy between RLT, immunotherapy, and targeted therapies, there is a growing optimism that these combined approaches will lead to more successful interventions in the battle against lung cancer, ultimately improving survival rates and quality of life for patients. The ongoing exploration of these strategies will be critical in establishing standardized protocols and determining the most effective combinations for various lung cancer subtypes.

### 6.3 Development of novel CXCR4-Targeting molecular probes

The development of novel CXCR4-targeting molecular probes represents a significant advancement in the field of molecular imaging, particularly in enhancing the sensitivity and specificity of imaging techniques used in oncology. Recent studies have focused on improving the imaging capabilities of radioligands such as 68Ga-Pentixafor, which targets the chemokine receptor CXCR4, known for its overexpression in various malignancies. Innovations in the synthesis of these probes aim to increase their binding affinity and retention in tumors, thus providing clearer imaging results. For instance, the introduction of cyclic peptides and modifications to existing ligands have shown promising results in preclinical models, where enhanced tumor uptake and reduced off-target effects were observed. These advancements are crucial as they allow for more precise localization of tumors, which is vital for effective treatment planning and monitoring. Furthermore, the integration of dual-targeting strategies, where probes are designed to bind to multiple tumor markers, is emerging as a trend in the field. Such multi-targeted imaging approaches could potentially provide a more comprehensive view of tumor biology and microenvironment, thereby improving diagnostic accuracy and therapeutic outcomes. Research comparing CXCR4-targeted radiotracers with [18F]FDG in head and neck squamous cell carcinoma has focused on diagnostic performance, without specific emphasis on CXCR4 as a therapeutic target (29).

#### 6.3.1 Enhancing imaging sensitivity and specificity wcith novel radioligands

The quest for enhanced imaging sensitivity and specificity has led to the development of new radioligands that target CXCR4, a receptor implicated in tumor progression and metastasis. These novel agents are designed to overcome the limitations of traditional imaging modalities, such as 18F-FDG PET, which may not adequately visualize tumors with low metabolic activity or those that do not express glucose transporters. For example, recent studies have demonstrated that 68Ga-Pentixafor PET can provide superior imaging contrast in malignancies characterized by high CXCR4 expression, such as multiple myeloma and certain lymphomas. By employing advanced labeling techniques and optimizing the pharmacokinetics of these radioligands, researchers have achieved significant improvements in tumor-to-background ratios, allowing for clearer delineation of tumor boundaries. Moreover, the development of hybrid imaging techniques that combine PET with other modalities, such as MRI, is also being explored to further enhance diagnostic capabilities. Studies have investigated splenic CXCR4 expression using [68Ga]Pentixafor PET in patients with solid tumors, without evidence supporting early tumor detection or treatment response monitoring (30).

#### 6.3.2 Trends in the development of multi-target imaging probes

The trend towards the development of multi-target imaging probes is gaining momentum in the field of molecular imaging, particularly for cancers expressing CXCR4. These innovative probes are designed to simultaneously target multiple biomarkers, thereby providing a more comprehensive assessment of tumor heterogeneity and microenvironment. For instance, researchers are exploring the conjugation of CXCR4-targeting ligands with other tumor-specific markers, which could enhance the sensitivity of imaging and improve the accuracy of tumor characterization. This approach is particularly beneficial in heterogeneous tumors where different regions may express varying levels of CXCR4 and other markers, complicating treatment decisions. By utilizing multi-target probes, clinicians can obtain a more nuanced understanding of the tumor’s biology, leading to tailored therapeutic strategies. Additionally, the integration of advanced imaging technologies, such as fluorescence imaging and nanotechnology, is being investigated to further enhance the specificity and efficacy of these multi-target probes. Advances in [68Ga]Pentixafor PET imaging provide valuable information for patient selection and prognosis in solid tumors, though claims of revolutionizing cancer diagnostics and personalized medicine exceed the scope of current evidence (31).

### 6.4 Radiomics and artificial intelligence-assisted diagnosis

The integration of radiomics and artificial intelligence (AI) into the diagnostic process of lung cancer, particularly through the analysis of PET/CT imaging data, represents a significant advancement in enhancing diagnostic accuracy and prognostic predictions. Radiomics involves the extraction of a large number of quantitative features from medical images, which can provide insights into tumor biology beyond what is visible to the naked eye. By employing deep learning techniques, researchers have been able to analyze these complex datasets to identify patterns that correlate with clinical outcomes. Artificial intelligence and radiomics have shown potential to enhance clinical decision-making in lung cancer, though definitive evidence of outperforming traditional diagnostic methods in sensitivity and specificity remains to be established (32). Moreover, AI-enhanced radiomics has shown promise in distinguishing between different histological subtypes of lung cancer, which is crucial for tailoring individualized treatment strategies. The ability of AI to learn from vast amounts of imaging data allows for the identification of subtle features that may be indicative of malignancy, thus facilitating more accurate staging and risk stratification (33). However, despite these advancements, challenges such as the need for standardized imaging protocols, reproducibility of radiomic features, and the necessity for large, diverse datasets for training AI models remain significant barriers to clinical implementation (34).

In addition to improving diagnostic accuracy, the integration of radiomics with molecular biomarkers has opened new avenues for personalized medicine in lung cancer management. By correlating radiomic features extracted from PET/CT scans with specific genetic and molecular profiles, researchers can gain insights into tumor behavior and treatment response. Artificial intelligence models integrating CT, PET, and clinical data have been developed to predict postoperative recurrence risk in early-stage non-small cell lung cancer, without specific focus on tumor microenvironment understanding (35). For example, studies have shown that certain radiomic features can predict the presence of specific mutations or the likelihood of response to targeted therapies, thereby enabling clinicians to tailor treatment plans based on individual patient profiles (36). Deep learning and CT-based radiomics analyses have been applied for automatic classification of lung cancer subtypes, without explicit evidence for identifying novel biomarkers beyond traditional histopathology (37). However, the successful integration of these technologies into clinical practice requires overcoming challenges related to data standardization, feature interpretability, and the need for extensive validation studies to ensure the robustness and generalizability of AI models across different populations and imaging modalities (38).

Overall, the application of radiomics and AI in lung cancer diagnosis represents a transformative shift towards more personalized and effective management strategies. As research continues to evolve, it is imperative to address the existing challenges and foster collaborations among researchers, clinicians, and technology developers to fully realize the potential of these innovations in improving patient outcomes in lung cancer care. The future of lung cancer diagnostics lies in the seamless integration of advanced imaging techniques, AI algorithms, and molecular data, paving the way for a more precise and individualized approach to cancer treatment (39).

### 6.5 Interdisciplinary collaboration promoting clinical translation

The integration of interdisciplinary collaboration is paramount in advancing the clinical application of imaging technologies like 68Ga-Pentixafor PET/CT in lung cancer management. Multidisciplinary teams, comprising oncologists, radiologists, nuclear medicine specialists, and researchers, work synergistically to bridge the gap between innovative imaging techniques and clinical treatment protocols. This collaboration not only enhances the diagnostic accuracy of 68Ga-Pentixafor PET/CT but also facilitates the development of tailored therapeutic strategies. For instance, studies have demonstrated that the CXCR4 receptor, which is overexpressed in various lung cancer subtypes, can be effectively targeted using 68Ga-Pentixafor PET imaging, allowing for real-time assessment of tumor biology and treatment response (4). By fostering a collaborative environment, clinical teams can leverage the strengths of each discipline to refine imaging protocols, improve patient selection for targeted therapies, and ultimately enhance patient outcomes. Furthermore, the combined expertise in molecular imaging and oncology can lead to the identification of novel biomarkers that predict treatment efficacy, thereby paving the way for personalized medicine approaches in lung cancer therapy.

In addition to enhancing diagnostic capabilities, interdisciplinary collaboration is essential for strengthening the bridge between basic research and clinical applications. The translation of findings from laboratory studies into clinical practice is often hindered by a lack of communication and understanding between researchers and clinicians. However, through collaborative efforts, researchers can gain insights into the clinical challenges faced by oncologists, which can inform their experimental designs and hypotheses. For example, the promising results of 68Ga-Pentixafor PET/CT in assessing CXCR4 expression in lung cancer highlight the need for robust clinical trials that validate these findings in diverse patient populations (22). By engaging in collaborative research initiatives, scientists can ensure that their work is aligned with clinical needs, thereby accelerating the pace of translational research. Additionally, joint efforts in education and training can equip healthcare professionals with the necessary skills to interpret advanced imaging results and incorporate them into clinical decision-making processes. This synergy between basic science and clinical application is crucial for developing innovative therapies that target the underlying mechanisms of lung cancer progression and resistance, ultimately leading to improved patient care and outcomes.

Moreover, interdisciplinary collaboration fosters an environment conducive to innovation, where diverse perspectives can lead to the development of novel imaging techniques and therapeutic strategies. The integration of advanced imaging modalities, such as 68Ga-Pentixafor PET/CT, with emerging therapeutic approaches, including CXCR4-targeted therapies, exemplifies how collaborative efforts can drive clinical advancements (23). By pooling resources and expertise, multidisciplinary teams can explore new avenues for treatment that were previously unconsidered, leading to breakthroughs in managing complex malignancies like lung cancer. As the landscape of cancer treatment continues to evolve, the importance of interdisciplinary collaboration cannot be overstated; it is essential for translating scientific discoveries into effective clinical applications that ultimately enhance patient outcomes and quality of life.

In conclusion, the promotion of interdisciplinary collaboration is vital for the successful clinical translation of 68Ga-Pentixafor PET/CT in lung cancer. By fostering teamwork among various specialties, healthcare professionals can enhance diagnostic accuracy, bridge the gap between research and clinical practice, and drive innovation in cancer treatment. This collaborative approach not only benefits individual patients but also contributes to the broader goal of advancing cancer care through the integration of cutting-edge imaging technologies and targeted therapies.

## 7 Conclusion

In conclusion, 68Ga-Pentixafor PET/CT is a promising advancement in the molecular imaging of lung cancer, especially for targeting the chemokine receptor CXCR4, which is notably overexpressed in small cell lung cancer (SCLC) and certain non-small cell lung cancer (NSCLC) subtypes. Clinically, this imaging modality offers a valuable tool for noninvasively assessing tumor biology, enabling more accurate staging, monitoring therapeutic response, and potentially guiding CXCR4-targeted radionuclide therapies. The ability of 68Ga-Pentixafor PET/CT to accurately reflect *in vivo* CXCR4 expression levels addresses a critical need in lung cancer management by providing functional insights beyond conventional anatomical imaging.

However, the current body of evidence is predominantly derived from small-scale, single-center studies, thus limiting the generalizability and robustness of the conclusions regarding its clinical utility. While enthusiasm generated by preliminary findings is understandable, rigorous validation through large-scale, multicenter clinical trials is imperative for future research. Such studies will be essential to confirm diagnostic accuracy, prognostic value, and therapeutic impact across diverse patient populations and lung cancer phenotypes. Moreover, standardized imaging protocols and quantification methods must be established to ensure reproducibility and facilitate integration into routine clinical workflows.

The evolving landscape of molecular imaging also presents opportunities to augment the capabilities of 68Ga-Pentixafor PET/CT. The development of novel radioligands with improved pharmacokinetics and higher affinity for CXCR4 could enhance image quality and diagnostic performance. Additionally, the incorporation of artificial intelligence (AI)-driven image analysis holds promise for more precise and automated interpretation, potentially uncovering subtle imaging biomarkers predictive of treatment response or resistance. Combining 68Ga-Pentixafor PET/CT with other imaging modalities, such as FDG PET/CT or MRI, may provide complementary information that refines tumor characterization and supports personalized therapeutic decision-making.

In synthesizing diverse research perspectives, it is clear that while 68Ga-Pentixafor PET/CT is not yet a standard component of lung cancer management, its integration into precision oncology paradigms is highly anticipated. The modality’s unique ability to visualize CXCR4 expression aligns with the broader trend toward biomarker-driven diagnosis and treatment. Realizing its full potential will require a multidisciplinary approach, encompassing radiochemistry, nuclear medicine, oncology, and computational sciences. Through collaborative efforts and well-designed clinical investigations, 68Ga-Pentixafor PET/CT could significantly impact patient outcomes by enabling tailored interventions that address tumor heterogeneity and dynamic biological processes.

Ultimately, the future of lung cancer care lies in the convergence of innovative imaging technologies and targeted therapies. 68Ga-Pentixafor PET/CT exemplifies this synergy, offering a pathway to more precise, effective, and individualized treatment strategies. Continued research and technological refinement will be vital to translating this promise into clinical reality, thereby improving prognosis and quality of life for patients afflicted with this challenging disease.
